# Supplementary material for: National health insurance membership in Indonesia: Do socio-economic elements matter?
Source: PLoS One. 2025 Aug 14;20(8):e0329781. doi: 10.1371/journal.pone.0329781 (PMC12352825; doi:10.1371/journal.pone.0329781)
Supplement: Attachment — Submitted filename: Answer to Reviewer_PLOS One_2309.docx [file pone.0329781.s001.docx]

**Answer to Reviewers**

**National health insurance membership in Indonesia: Does education level matter?**

| **No** | **Reviewers** | **Reviewer Comments** | **Answer to Reviewer** |
| --- | --- | --- | --- |
| 1 | Reviewer 1 | In my opinion, the relationship between education level and insurance membership is not a meaningful research topic worth pursuing as there is no evidence or knowledge gap for this issue. The author provided a comprehensive summary on the factors that impacted health insurance membership in Introduction and it is clear that there is no new information in this study that can contribute to literature. | Existing literature has extensively covered various factors affecting health insurance membership, but there is still limited evidence regarding how education levels interact with other socioeconomic factors in specific contexts. Our study contributes by exploring this interaction in a novel setting, offering insights that could inform policy interventions aimed at improving insurance coverage among underserved populations.  Moreover, while the introduction of our manuscript provides a comprehensive summary of known factors, the primary goal was to establish the foundation upon which our research question is built. We believe that our findings, which highlight the importance of education in insurance uptake, add value to the existing body of knowledge, particularly in regions where educational disparities significantly impact access to health services. |
| 2 | Reviewer 2 | Improve the first paragraph by establishing a context that emphasizes the importance of discussing health insurance issues from a global perspective. Then, continue with a more specific explanation regarding the reasons why research on health insurance in Indonesia is very important.  Following the statement of purpose, it is important to highlight the potential benefits that can be derived from the results of this research. These benefits can range from informing policymaking to increasing the effectiveness of health insurance programs. | Thank you for your feedback, please see the revised in the **Line 45 - 59:**  “In the global landscape, health insurance is increasingly recognized as a cornerstone of sustainable development, influencing not only individual well-being but also broader economic and social outcomes. Across various countries, the implementation of comprehensive health insurance programs has been linked to improved public health, reduced poverty, and more equitable access to essential services. As nations strive to achieve universal health coverage (UHC), the effectiveness of health insurance schemes becomes a critical focus for policymakers and researchers alike. In this global context, examining health insurance in Indonesia is particularly important due to the unique challenges and opportunities presented by its diverse population and rapidly evolving healthcare system. The National Health Insurance (NHI) Program launched as a pivotal state initiative, has demonstrated tangible positive impacts on economic growth and enhanced access to healthcare services for millions of Indonesians [1]. It embodies the government's commitment to safeguarding the fundamental rights of its citizens [2] by ensuring equal and equitable access to healthcare. Understanding the dynamics of this program within Indonesia contributes to the global discourse on UHC. It provides valuable insights into the specific strategies that can be employed to strengthen health systems in similar contexts.”  **Line 127-141:**  “The potential benefits derived from this research are substantial. By examining the role of education level on NHI membership in Indonesia, the findings can provide crucial insights that inform more targeted and effective policymaking. Specifically, understanding how education influences health insurance uptake can help policymakers design interventions that better address the disparities in coverage, particularly in underserved regions like eastern Indonesia [19]. These insights could guide the allocation of resources, ensuring that investments in health infrastructure and human resources are directed where they are most needed, thereby reducing inequalities between provinces [20,21]. Additionally, the research findings could support the development of health promotion strategies that are tailored to different educational levels, making health insurance programs more accessible and understandable to all segments of the population.  Moreover, this research has the potential to increase the overall effectiveness of the NHI Program by highlighting the importance of integrating educational initiatives with insurance enrollment efforts. This could lead to higher participation rates, especially among low-income and less-educated communities, ultimately contributing to greater health equity across the country. By addressing these gaps, the study supports the broader goal of achieving universal health coverage in Indonesia, ensuring that every citizen, regardless of their educational background, has access to the healthcare services they need.” |
|  |  | Methods and results The outcome variable in this research is membership in Indonesia's national health insurance. As is known, national health insurance membership in Indonesia is divided into several types, consisting of Contribution Assistance Recipients (PBI in Indonesian), Wage Recipient Workers (PPU), Non-Wage Recipient Workers (PBPU in Indonesian), and Non-Workers (PB in Indonesian). Why doesn't the author differentiate National Health Insurance membership based on this scheme? Can data analysis be carried out taking into account the existence of several of these schemes? If the author has reasons, explain clearly why the data analysis design ignores the types of JKN participation mentioned above. | Thank you for your thoughtful feedback. We acknowledge the importance of distinguishing between the different types of National Health Insurance (NHI) membership in Indonesia, as each category—Contribution Assistance Recipients (PBI), Wage Recipient Workers (PPU), Non-Wage Recipient Workers (PBPU), and Non-Workers (PB)—represents distinct socioeconomic groups with varying access to healthcare services.  However, in this study, our primary objective was to explore the general relationship between education level and overall NHI membership, rather than focusing on the specific differences between these membership types. By analyzing NHI membership in aggregate, we were able to capture the general influence of education on health insurance uptake without the confounding effects that might arise from the specific characteristics of each membership type. This approach allowed us to provide a clearer, more generalizable insight into how education impacts health insurance enrollment across diverse population groups.  We recognize the value of examining the specific differences between NHI membership types, and this could indeed be a fruitful area for future research. Future studies could benefit from a more granular analysis if more detailed and comprehensive data becomes available. We believe that such research would complement our current findings by providing a more nuanced understanding of how different socioeconomic factors influence health insurance participation in Indonesia. |
|  |  | Discussion In line 266, the author states the relationship between educational-level access to information and health literacy. Apart from these two things, there is a possibility that education is also related to work and welfare, as quoted from research in India. What about research in Indonesia? Add discussion about this. | Thank you for your feedback, please see the revised in the **Line 304 - 310:**  “In the Indonesian context, education is not only linked to better access to information and improved health literacy, but it also plays a crucial role in shaping employment opportunities and overall economic welfare. Higher levels of education are strongly associated with better job prospects, higher income, and improved living standards. This relationship is supported by several studies conducted in Indonesia, which have shown that individuals with higher education levels are more likely to secure formal employment, earn higher wages, and experience greater economic security (Laksono 2022, Rahmawati 2024).” |
| 3 | Reviewer 3 | General comment: The authors examined the role of education level on NHI membership in Indonesia. Using a cross-sectional design, this study examined 1,223,377 individuals using the latest secondary data from the 2023 National Socioeconomic Survey showing fluctuations in NHI participation since 2021. Authors provided justification the reason of fluctuations indicating Various factors contribute to this shortfall, notably compliance enforcement, particularly among economically vulnerable demographics. A comprehensive approach encompassing education, legal enforcement, and incentives is imperative to ensure a universal understanding of the significance and obligations associated with NHIP participation. | Thank you for your insightful feedback. Our study found that education level plays a significant role in NHI participation, particularly in shaping individuals' understanding and awareness of the importance of health insurance. Individuals with higher education levels are more likely to be informed about the benefits of NHI and the obligations associated with participation. This awareness translates into higher enrollment rates among those with more education.  However, we also observed that education alone is not sufficient to ensure widespread participation. The fluctuations in NHI membership can be attributed to various factors, including the challenges of compliance enforcement, especially among economically vulnerable groups who may face barriers to accessing information and resources. Therefore, we propose that a multifaceted approach is necessary to address these issues. |
|  |  | 1. Authors focus of interest was contribution of education level to public awareness to participate in NHI membership served as the outcome variable  Comment: the article focused on contribution of education level on public awareness to participate in NHI independently and voluntarily. Please elaborate what the authors mean with public awareness. Is it in the level of awareness or the level of participation. Authors need to justify choosing level of education on NHI participation as variable of interest. As most study identified those with high education level has better health status compared to those with lower education. The authors pointed out that wealthier group have greater chance to be NHI member, which is common in most countries that those with better education and economic status is more concerned on their health, including NHI participation. What is the specific condition of Indonesian NHI compared to NHI in other countries which make the authors pay specific interest on analysis contribution of education level on NHI participation.  "The authors should clarify if they are examining the contribution of education level to public awareness of NHI benefits, or actual participation in the program." | The focus of this study is on understanding the contribution of education level to both public awareness and actual participation in Indonesia's National Health Insurance (NHI) program. When we refer to "public awareness," we mean the awareness of the benefits and obligations associated with NHI, which includes understanding the importance of health insurance and the steps required to enroll in and utilize the program. However, our primary outcome variable is actual participation in NHI, which is influenced by this awareness.  We selected education level as a key variable because education plays a critical role in shaping an individual's health literacy, including their ability to understand and engage with health insurance programs. Higher education is generally associated with better health outcomes and a greater likelihood of participating in health programs like NHI. This is because individuals with higher education levels typically have better access to information, are more likely to understand the benefits of NHI, and are more proactive about their health.  In Indonesia, the context is unique due to significant disparities in education, income, and access to healthcare across different regions and population groups. Unlike in many other countries where health insurance participation is more evenly distributed, in Indonesia, these disparities create significant barriers to achieving universal health coverage. The wealthier and more educated populations are more likely to participate in NHI, leaving the socioeconomically disadvantaged at a disadvantage.  The specific interest in education level in this study arises from the hypothesis that increasing educational attainment could significantly enhance NHI participation, particularly among disadvantaged groups. In Indonesia, where the NHI program is relatively new and faces challenges in reaching universal coverage, understanding the role of education can provide valuable insights for policymakers. The goal is to identify strategies that can increase participation rates by improving public awareness and reducing barriers to enrollment, particularly for those with lower education levels. |
|  |  | 2. Authors admit that Education plays a crucial role in influencing health insurance ownership and utilization. Education level has a significant impact on health insurance ownership. A study conducted in Indonesia found that pregnant women with higher education were 3.349 times more likely than 86 those with no education to have health insurance. Another study in China found that education significantly increased the demand for commercial health insurance. Furthermore, research conducted on 26 OECD countries from 1995 to 2015 found that adults with higher educational attainment better understand and appreciate health insurance's benefits, thereby increasing ownership rates. Education improves access to resources, including health insurance, which enables individuals to receive better healthcare services. Additionally, educated individuals are more likely to engage in healthy behaviors and recognize the importance of health insurance.  Since contribution of education on NHI membership is the authors main interest, please provide more justification why selecting the variable. Authors also admit the contribution of other demographic factors on NHI participation. Please elaborate how the intertwined of education level with other demographic factors on NHI participation.  "While the link between education and health insurance is established, the authors should elaborate on why education is a specific interest here. Do they expect a different dynamic in Indonesia's NHI compared to other countries?" | The selection of education level as a key variable in examining NHI membership in Indonesia is grounded in its fundamental role in shaping individuals' understanding of health-related issues, including the importance and benefits of health insurance. Education is not only a determinant of health literacy but also influences an individual's ability to navigate complex healthcare systems, understand the benefits of insurance, and make informed decisions regarding health coverage. In Indonesia, where disparities in education levels are significant, the role of education becomes even more critical in determining NHI participation.  While the relationship between education and health insurance participation is well-established globally, the dynamics in Indonesia may differ due to the unique challenges the country faces, such as socioeconomic disparities, geographic barriers, and varying levels of access to information. The National Health Insurance program in Indonesia aims to provide universal coverage, yet participation rates vary widely, often correlating with education levels. Influences widespread educational inequality in Indonesia, understanding how education influences NHI membership is crucial for designing effective interventions to increase participation.  We’ve add  Additionally, education does not operate in isolation; it is closely intertwined with other demographic factors such as income, employment status, residence, and age. For instance, higher education levels are often associated with better employment opportunities and higher income, which can, in turn, increase the likelihood of NHI participation. Furthermore, individuals with higher education may be more likely to live in urban areas where access to healthcare services and information about NHI is greater. The interaction of these factors creates a complex web that influences NHI membership. In the  By focusing on education, the study aims to uncover these intricate relationships and provide insights into how improving educational opportunities could lead to higher NHI participation, particularly among disadvantaged groups. The authors hypothesize that while education's impact on NHI membership is likely to align with global trends, the specific context of Indonesia—characterized by significant regional, socioeconomic, and educational disparities—may present unique challenges and opportunities that warrant a focused analysis of this variable. |
|  |  | 3. Table 1: please add total N in the left column of NHI membership, with column percent to make reader understand the big picture of population according to each explanatory variable. | Thank you for your suggestion. We agree that including the total number (N) in the left column of the NHI membership, along with column percentages, will provide a clearer understanding of the population distribution across the explanatory variables. We have revise Table 1 to include the total N for each category of NHI membership and provide the corresponding column percentages. |
|  |  | 4. How is the strength of the model as shown in the classification table? How the authors explain the strength of the model to increase NHI participation level? | Thank you for your question. To evaluate the strength of the logistic regression model in Table 2, we interpret the model's effectiveness based on the Adjusted Odds Ratios (AORs) and their significance. The AORs indicate the likelihood of NHI membership across different predictor variables after adjusting for other factors. For example, individuals with a college education are nearly twice as likely to be NHI members compared to those with only primary education, as reflected by an AOR of 1.916. The statistically significant p-values (all < 0.001) suggest that these predictors are strong and reliable. Although the classification table is not shown, if the model demonstrates high classification accuracy, it would indicate strong predictive power, effectively distinguishing between NHI members and non-members. The narrow confidence intervals around the AORs further imply precise estimates, reinforcing the model’s reliability.  The model’s strength, demonstrated by these significant and robust relationships, provides actionable insights for increasing NHI participation. The strong association between higher education levels and NHI membership suggests that improving education and health literacy could be an effective strategy, particularly for those with lower education levels. Furthermore, the lower odds of NHI membership among rural and economically disadvantaged groups highlight the need for targeted interventions, such as subsidies, incentives, and outreach programs, to make NHI more accessible in these areas. The significant associations with age, gender, and marital status indicate that addressing specific demographic needs, such as tailored programs for older adults or women, could further enhance participation. Additionally, the positive relationship between employment, wealth, and NHI membership suggests that improving economic conditions and linking NHI enrollment to employment benefits could naturally lead to higher participation rates. |
|  |  | 5. Line 192: authors gave figure on the hospital utilisation. Please explain how the association of the figure with the topic of the manuscript which is contribution of education on NHI membership. "It would be helpful to understand how the hospital utilization figure connects to the main topic of education and NHI participation. Is it presented as a potential benefit of NHI membership?" | We decided to skip this figure from the manuscript. |
|  |  | 6. Line 258 – 260: please elaborate the meaning of the sentence some previous studies showed the same results that Higher education is associated with a higher likelihood of NHI membership among the poor. Did authors mean poor people with higher education more likely to participate in NHI?  "This sentence could be rephrased for clarity. Did the authors find that even among low-income individuals, those with higher education were more likely to participate in NHI?" | Thank you for your suggestion. Please see the revised in **Line 277-291:**  “The result showed that education level is related to NHI membership in Indonesia. The results of this study indicate that education has a dominant influence on NHI participants in Indonesia. This is in line with research in Indonesia on a special population involving more than a thousand underprivileged people, which found that those who were probable to become NHI participants, compared to those who were educated at the middle and elementary school levels [14,30]. Indonesia has made commendable progress in expanding UHC through its NHI mechanism. However, socioeconomic disparities persist, leading to varying levels of understanding of NHI concepts and procedures among different subpopulations [31]. These disparities can impact healthcare access equity.  Previous studies have found similar results, indicating that individuals with higher levels of education are more likely to participate in NHI programs, even within low-income groups [14]. This means that poor individuals with higher levels of education are more likely to enrol in NHI. This trend is often attributed to better awareness of healthcare options, a greater understanding of the benefits of health insurance, and potentially higher income levels are more common among those with higher education, all of which facilitate NHI enrollment [32–35].” |
|  |  | 7. The analysis showed that higher education and wealthiest are more likely to participate on NHI than those with lower education and least wealthy. How is the proportion of higher education and wealthiest among Indonesian compared to those with lower education and least wealthy? As authors said those with higher education and wealthier is more exposed to benefit of NHI. What authors recommendation for the government for people with lower education, least wealthy, less exposure to get NHI benefit. | The analysis shows that individuals with higher education and greater wealth are more likely to participate in the National Health Insurance (NHI) program compared to those with lower education and less wealth. In Indonesia, the proportion of people with lower education and lower wealth is significantly larger than those with higher education and greater wealth, presenting a challenge for equitable NHI coverage. Given that higher-educated and wealthier individuals are more exposed to and likely to understand the benefits of NHI, it is crucial to focus on strategies to increase participation among the less educated and economically disadvantaged groups. The government should implement targeted health literacy programs to educate those with lower education levels about NHI benefits, using community outreach, mass media, and partnerships with local leaders to ensure broad reach. Financial barriers can be addressed by subsidizing or fully covering NHI premiums for the poorest populations, making NHI more accessible. Simplifying the NHI enrollment process and ensuring it is easily accessible, particularly for those in rural areas or with limited education, is also essential. Additionally, leveraging community-based health programs and integrating NHI awareness into school curricula can help bridge the gap for those with less exposure to NHI benefits. These strategies can contribute to a more inclusive NHI system that ensures everyone has access to health insurance benefits regardless of education level or wealth.  Please see the revision in **Lines 369-384** |
|  |  | 8. Line 289 – 293: Socioeconomically disadvantaged communities living in rural areas in Indonesia face a variety of barriers that limit their access to primary health care; this is the government's primary concern, including the availability of a sufficient number and quality of health workers, combined with a telemedicine approach, to ensure the availability of health services and a reference guide:  Comment: what authors intend to say Socioeconomically disadvantaged communities living in rural areas in Indonesia face a variety of barriers that limit their access to primary health care with the telemedicine approach? Did authors intend to say telemedicine approach as solution for the socioeconomically disadvantaged communities living in rural areas? o "The authors' discussion of telemedicine as a solution for rural communities with NHI access limitations needs clarification. Is telemedicine presented as a potential solution to improve access for these communities?" | The intention of the authors was indeed to highlight telemedicine as a potential solution to improve healthcare access for socioeconomically disadvantaged communities in rural areas of Indonesia. These communities often face significant barriers to accessing primary healthcare services, such as a shortage of healthcare providers, long distances to healthcare facilities, and limited transportation options. Telemedicine can help bridge this gap by providing remote access to healthcare services, enabling patients to consult with healthcare professionals without the need to travel.  The government’s focus on combining telemedicine with efforts to increase the availability and quality of health workers in rural areas aims to ensure that even the most underserved populations can receive timely and adequate healthcare. Telemedicine is thus presented as part of a broader strategy to overcome the healthcare access limitations faced by rural communities, particularly within the framework of the National Health Insurance (NHI) program. |
|  |  | 9. Line 294 – 295: did author mean living in rural as barrier for NHI participation? | Please see the revised in **Lines 323-332:**  “Socioeconomically disadvantaged communities in rural areas of Indonesia face significant barriers to accessing primary healthcare, which impacts their participation in the NHI program. These barriers include a shortage of healthcare facilities and providers, as well as logistical challenges like long-distance and inadequate transportation options, making it difficult for rural residents to enrol in NHI, even if they understand its benefit[7] [44]. Additionally, cultural beliefs that emphasize self-reliance and the use of home remedies, coupled with skepticisms toward government institutions, further diminish the perceived need for health insurance in these communities. These factors contribute to lower NHI enrollment rates in rural areas, consistent with findings from a meta-analysis of 12 publications, which identified rural residency as a significant factor influencing NHI ownership in Indonesia.” |
|  |  | 10. Authors provided comprehensive explanation on the dynamics of NHI participation | Thank you for your suggestion |
