# Supplementary material for: National health insurance membership in Indonesia: Do socio-economic elements matter?
Source: PLoS One. 2025 Aug 14;20(8):e0329781. doi: 10.1371/journal.pone.0329781 (PMC12352825; doi:10.1371/journal.pone.0329781)
Supplement: Attachment — Submitted filename: ANSWER TO EDITOR_PLOSONE_2ND_0412.docx [file pone.0329781.s002.docx]

ANSWER TO EDITOR

| **No** | **Reviewers** | **Reviewer’s Comments** | **Answer to Reviewer** |
| --- | --- | --- | --- |
| 1 | Journal requirements | Please review your reference list to ensure that it is complete and correct. If you have cited papers that have been retracted, please include the rationale for doing so in the manuscript text, or remove these references and replace them with relevant current references. Any changes to the reference list should be mentioned in the rebuttal letter that accompanies your revised manuscript. If you need to cite a retracted article, indicate the article’s retracted status in the References list and also include a citation and full reference for the retraction notice. | Thank you for highlighting this important issue. We carefully reviewed the reference list to ensure its completeness, accuracy, and alignment with current academic standards. Below are the steps we took and corresponding actions:   1. Verification of References:  - We cross-checked all references against reputable databases (e.g., Scopus, PubMed, and Web of Science) to verify their status. - No retracted articles were identified in the current reference list.  2. Completeness Check:  - We ensured all references were cited appropriately in the text. Missing references were added, and redundant or unrelated references were removed. |
| 2 | Editor | ‘National health insurance membership in Indonesia: Do socio-economic elements matter’ may be considered more appropriate for the manuscript considering the employment of socio-economic variables such as education, wealth, et cetera (NOT ONLY EDUCATION, ALTHOUGH EDUCATION MAY HAVE ‘DOMINANT INFLUENCE’). | Thank you for suggesting revising the manuscript title to represent better the scope of socio-economic variables used in the study. We agree that the current title may not fully capture the factors examined. While education is highlighted for its dominant influence, the analysis also incorporates variables such as wealth and other socioeconomic factors.  Revised Title:  National health insurance membership in Indonesia: Do socio-economic elements matter |
| 3 |  | Conclusions and recommendations must emanate from findings. For examples, “…COMMUNITY WELFARE provide more opportunities for individuals to engage in NHI” AND “…targeted health literacy initiatives should focus on INFORMAL SECTOR WORKERS…” were not part of the findings. Many more conclusions can be drawn from the findings and more recommendations can be made without exaggeration. Authors should maximize the use of this section to benefit readers and policy makers in a concise manner. For instance, since wealth influenced NHI membership, subsidy/premium waiver for the less-privileged may be more appropriate as recommendation, among others. | Thank you for this constructive feedback. We revised the Conclusions and Recommendations section to ensure the findings directly support all points. We have also expanded recommendations to include specific, actionable strategies based on the results, particularly addressing wealth disparities.  Revised Conclusions and Recommendations Section:  Conclusions  This study highlights the significant role of education level in influencing NHI membership in Indonesia. Individuals with higher education levels are more likely to enrol, as education improves health literacy and awareness of health insurance benefits. Wealthier individuals and those in urban areas also show higher enrollment rates, indicating persistent socioeconomic and geographic disparities. These findings underscore the need for targeted strategies to address barriers to NHI membership among disadvantaged groups, particularly in rural areas and among low-income populations.  Recommendations  1. Addressing Wealth Disparities:  To mitigate the impact of wealth on NHI membership, the government could consider introducing premium waivers or subsidies for low-income groups. Such measures would reduce financial barriers, particularly for individuals in the poorest wealth quintiles.  2. Health Literacy Campaigns:  Targeted health literacy initiatives should focus on enhancing awareness about the benefits of NHI, especially among groups with lower education levels. Community-based programs could be tailored to informal sector workers, leveraging local leaders and trusted institutions to deliver health education effectively.  3. Improving Accessibility in Rural Areas:  Policies should prioritize digital and physical infrastructure development in rural regions to improve access to NHI registration and healthcare services. Mobile registration units and partnerships with local governments could address geographic barriers.  4. Integrating Education with Health Policy:  The findings underscore the importance of integrating educational initiatives with health insurance outreach programs. For instance, school-based campaigns and adult education programs could include modules on health literacy and the benefits of NHI enrollment.  5. Enhancing Policy Implementation:  Policymakers should strengthen cross-sectoral collaboration to implement these recommendations effectively. Coordination among the healthcare, education, and social welfare sectors is essential to address the multifaceted barriers to NHI enrollment. |
