# Supplementary material for: National health insurance membership in Indonesia: Do socio-economic elements matter?
Source: PLoS One. 2025 Aug 14;20(8):e0329781. doi: 10.1371/journal.pone.0329781 (PMC12352825; doi:10.1371/journal.pone.0329781)
Supplement: Attachment — Submitted filename: Comment_jan2025.docx [file pone.0329781.s003.docx]

**Thank you for your excellent work in addressing and clarifying the reviewer inputs. Below are my comments:**

General comment

The title National health insurance membership in Indonesia: Do socio economic elements matter? However, in abstract authors seem to focus more on impact of education level on NHI membership.

Specific comments

**Abstract**

Line 27 – 29

The study examined the role of education level on NHI membership in Indonesia. Using a cross-sectional design, this study examined 1,223,377 individuals. Level of education was utilized as the exposure variable, while NHI membership served as the outcome variable

Comment

What is the objective of the study? Examine role of education level on NHI membership?

Results showed that compared to those with primary education, higher education have higher odds for NHI membership. Authors, please make brief sentences.

**Introduction result and discussion**

Line 105 - 108

While previous studies have examined factors influencing NHI membership in Indonesia, limited research has focused specifically on the role of education level, particularly among the poor and disadvantaged groups. This study aims to fill this gap by analysing how educational attainment influences NHI membership across different socioeconomic strata.

Comment

Author objective is to identify different contribution of education across socioeconomic strata on NHI membership. However, results from table 3 did not show different contribution of education on NHI membership across economic strata. Table 3 indeed showed contribution of each exposure variables on NHI membership

**Great job overall!**
