# Supplementary material for: National health insurance membership in Indonesia: Do socio-economic elements matter?
Source: PLoS One. 2025 Aug 14;20(8):e0329781. doi: 10.1371/journal.pone.0329781 (PMC12352825; doi:10.1371/journal.pone.0329781)
Supplement: Attachment — Submitted filename: Comments.docx [file pone.0329781.s004.docx]

**National health insurance membership in Indonesia: Do socio-economic elements matter?**

The authors have been able to attend to the comments well in ‘response to editor’s comment section’. However, I was wondering if the authors effected the corrections in the manuscript submitted for review. Specifically, there seems not to be any difference between the conclusion in Revision 2 and Revision 1 as shown below:

**R2 Conclusion**

The study findings reveal a strong correlation between education level and participation in NHI program. Individuals with higher education levels and greater community welfare are more likely to engage in NHI. To increase participation, especially in rural areas with limited public information, targeted health literacy initiatives should focus on informal sector workers, the younger generation, and singles. These initiatives can emphasize the importance of preventive health measures and the benefits of NHI in improving productivity. Through cross-sector cooperation with central and regional stakeholders, the government can also work to establish universally available primary health services for all citizens. The study further concludes that wealthier individuals are more likely to join NHI. To expand coverage, the government should prioritize low-income groups and remote areas by simplifying the registration process. This could involve offering online registration, deploying mobile registration units to reach rural communities, and providing clear eligibility guidelines. Public awareness campaigns should educate citizens on the benefits of NHI, the enrollment process, and available services. Additionally, ensuring that healthcare services covered by NHI meet quality standards is crucial. This effort should include regular monitoring, patient feedback mechanisms, and incentives for providers to maintain high-quality care. Future research should explore NHI membership by disaggregating data based on the type of insurance scheme. Such studies could provide more detailed insights into the effectiveness of specific insurance models in reaching underserved populations and inform targeted policy interventions to improve coverage equity.

**R1 Conclusion**

The study findings reveal a strong correlation between education level and participation in NHI program. Individuals with higher education levels and greater community welfare are more likely to engage in NHI. To increase participation, especially in rural areas with limited public information, targeted health literacy initiatives should focus on informal sector workers, the younger generation, and singles. These initiatives can emphasize the importance of preventive health measures and the benefits of NHI in improving productivity. Through cross-sector cooperation with central and regional stakeholders, the government can also work to establish universally available primary health services for all citizens. The study further concludes that wealthier individuals are more likely to join NHI. To expand coverage, the government should prioritize low-income groups and remote areas by simplifying the registration process. This could involve offering online registration, deploying mobile registration units to reach rural communities, and providing clear eligibility guidelines. Public awareness campaigns should educate citizens on the benefits of NHI, the enrollment process, and available services. Additionally, ensuring that healthcare services covered by NHI meet quality standards is crucial. This effort should include regular monitoring, patient feedback mechanisms, and incentives for providers to maintain high-quality care. Future research should explore NHI membership by disaggregating data based on the type of insurance scheme. Such studies could provide more detailed insights into the effectiveness of specific insurance models in reaching underserved populations and inform targeted policy interventions to improve coverage equity.

**Conclusion: Original submission**

The study findings reveal a strong correlation between education level and participation in NHI program. Individuals with higher education levels and greater community welfare are more likely to engage in NHI. To increase participation, especially in rural areas with limited public information, targeted health literacy initiatives should focus on informal sector workers, the younger generation, and singles. These initiatives can emphasize the importance of preventive health measures and the benefits of NHI in improving productivity. Through cross-sector cooperation with central and regional stakeholders, the government can also work to establish universally available primary health services for all citizens. The study further concludes that wealthier individuals are more likely to join NHI. To expand coverage, the government should prioritize low-income groups and remote areas by simplifying the registration process. This could involve offering online registration, deploying mobile registration units to reach rural communities, and providing clear eligibility guidelines. Public awareness campaigns should educate citizens on the benefits of NHI, the enrollment process, and available services. Additionally, ensuring that healthcare services covered by NHI meet quality standards is crucial. This effort should include regular monitoring, patient feedback mechanisms, and incentives for providers to maintain high-quality care.

Additionally, and as noted by the reviewer, authors must modify all sections in the manuscript to reflect the new title.

Thank you.
