# Supplementary material for: National health insurance membership in Indonesia: Do socio-economic elements matter?
Source: PLoS One. 2025 Aug 14;20(8):e0329781. doi: 10.1371/journal.pone.0329781 (PMC12352825; doi:10.1371/journal.pone.0329781)
Supplement: Attachment — Submitted filename: ANSWER TO EDITOR_PLOSONE_3rd_2204.docx [file pone.0329781.s005.docx]

ANSWER TO EDITOR

| **No** | **Reviewers** | **Reviewer’s Comments** | **Answer to Reviewer** |
| --- | --- | --- | --- |
| 1 | Journal requirements | Please review your reference list to ensure that it is complete and correct. If you have cited papers that have been retracted, please include the rationale for doing so in the manuscript text, or remove these references and replace them with relevant current references. Any changes to the reference list should be mentioned in the rebuttal letter that accompanies your revised manuscript. If you need to cite a retracted article, indicate the article’s retracted status in the References list and also include a citation and full reference for the retraction notice. | We would like to thank the Editor for the valuable suggestion regarding the review and revision of our reference list. Following a thorough cross-check, we identified that Reference No. 14 and Reference No. 30 refer to the same source. “  “ Putri NK, Laksono AD, Rohmah N. Predictors of National Health Insurance Membership among The Poor with Different Education Levels in Indonesia. BMC Public Health 2023;23:373. https://doi.org/10.1186/s12889-023-15292-9.  This duplication occurred inadvertently during the revision process as a result of the merging of content from earlier manuscript versions.  To address this, we have removed Reference No. 30 and retained Reference No. 14 as the correct and singular citation for this source. All in-text citations that previously referred to Reference No. 30 have been updated accordingly to maintain consistency and accuracy throughout the manuscript.  We trust that this amendment satisfies the requirement for a complete and accurate reference list, and we appreciate the opportunity to clarify this matter.  We have added links of the DOI in the references below:   - [12] Wulandari RD, Laksono AD, Rohmah N, Ashar H. Regional Differences in Primary Healthcare Utilization in Java Region—Indonesia. PLoS One 2023;18:e0283709-. [**https://doi.org/10.1371/journal.pone.0283709**](https://doi.org/10.1371/journal.pone.0283709)**.** - [13] Skinner EH, Foster M, Mitchell G, Haynes M, O’Flaherty M, Haines TP. Effect of health insurance on the utilisation of allied health services by people with chronic disease: a systematic review and meta-analysis. Aust J Prim Health 2014;20:9–19**.** [**https://doi.org/10.1071/PY13092**](https://doi.org/10.1071/PY13092). - Oldfisra F, Machdum SV. Analisis Perencanaan Kebijakan Jaminan Kesehatan Nasional dari Aspek Kepesertaan di Kementerian PPN/Bappenas. Jurnal Ilmu Kesejahteraan Sosial 2020;21:2656–9604. [**https://doi.org/10.7454/jurnalkessos.v21i1.253**](https://doi.org/10.7454/jurnalkessos.v21i1.253). - Bela Sari, Haerawati Idris. Determinant of Independent National Health Insurance Ownership in Indonesia. Malaysian Journal of Public Health Medicine 2019;19:109–15. [**https://doi.org/10.37268/mjphm/vol.19/no.2/art.177**](https://doi.org/10.37268/mjphm/vol.19/no.2/art.177).   We have added the English version for the Indonesian reference title:   - Herawati, Franzone R, Chrisnahutama A. Universal Health Coverage: Mengukur Capaian Indonesia **(Universal Health Coverage: Measuring Indonesia’s Achievements).** 2020. |
| 2 | Editor | The authors have been able to attend to the comments well in ‘response to editor’s comment section’. However, I was wondering if the authors effected the corrections in the manuscript submitted for review. Specifically, there seems not to be any difference between the conclusion in Revision 2 and Revision 1. | We thank the Editor for the careful reading of our revised manuscript and for bringing attention to the conclusion section. We sincerely apologize for the oversight in the earlier submission. After reviewing Revision 1 and Revision 2, we acknowledge that the Conclusion section in both versions was largely similar, and insufficient adjustments had been made to reflect the expanded and revised discussion as recommended in earlier reviews.  We have now thoroughly revised the Conclusion section in the current version of the manuscript (Line 381). The updated conclusion draws directly from the findings and discussion, specifically highlighting:  1. The dominant role of education in shaping NHI membership across different socioeconomic groups;  2. The influence of wealth status, residence, and demographic variables (age, gender, marital status, and employment);  3. The actionable policy implications derived from these findings.  The revised version now better aligns with the recommendations and empirical results presented throughout the paper.  “This study demonstrates that educational attainment plays a pivotal role in determining enrolment in Indonesia’s National Health Insurance (NHI) programme. Individuals with higher levels of education are significantly more likely to be NHI members, which may be attributed to greater health literacy, better understanding of insurance benefits, and enhanced access to relevant information. In addition to education, other socio-economic and demographic variables—including wealth status, residence (urban or rural), age, sex, marital status, and employment—were also found to be statistically significant predictors of NHI membership.  The findings indicate that individuals residing in rural areas, those with lower educational levels, the unemployed, and members of the lowest wealth quintiles are less likely to be enrolled in the NHI. This underscores persistent disparities in access to social protection and suggests the need for more equitable outreach and policy intervention.  To improve NHI coverage and ensure that the goal of universal health coverage (UHC) is realised, particular attention must be directed towards these underserved populations. Policy responses should include strengthening community-based health literacy programmes, simplifying administrative enrolment procedures, offering premium waivers or targeted subsidies for low-income groups, and enhancing the accessibility of primary healthcare services across all regions, particularly in rural and remote areas.” |
| 3 |  | Additionally, and as noted by the reviewer, authors must modify all sections in the manuscript to reflect the new title. | We thank the editor for this important note. The manuscript has been revised accordingly to reflect the updated title:  “National health insurance membership in Indonesia: Do socio-economic elements matter?”  Previously: “Does education level matter?”  We systematically revised all sections of the manuscript to align with the broader scope implied by the new title, shifting emphasis from education alone to multiple socioeconomic elements. Below are the section-specific adjustments:   - Modified sentence in Abstract (Line 25-42):   "The Indonesian government released a National Health Insurance (NHI) policy to realize universal health coverage (UHC) in Indonesia**, yet disparities in membership persist. The study examined the influence of various socio-economic factors, including education, employment, and wealth status, on National Health Insurance (NHI) membership in Indonesia.** Using a cross-sectional design, this study examined 1,223,377 individuals. Level of education was utilized as the exposure variable, while NHI membership served as the outcome variable. The analysis included residence, age, gender, marital status, and occupation as covariates. In the final stage, logistic multinomial regression was implemented. Based on the findings of this study, Indonesia’s coverage for NHI membership in 2023 was 72.5%. Regarding education level, the result indicates that junior high school is 1.214 times more likely to be an NHI member than primary school 95% CI 1.200-1.228). Meanwhile, senior high school is 1.301 times more likely than primary school to be an NHI member (95% CI 1.286-1-1.315). Moreover, college is 1.916 times more likely to own NHI than primary school (95% CI 1.878-1.954). Wealth status and demographic factors such as age and marital status also showed significant associations with NHI membership. According to the results, the study concluded that education level and wealth status significantly influence NHI membership in Indonesia. Demographic factors also play essential roles. Tailored interventions addressing these factors are necessary to improve NHI enrollment and achieve universal health coverage."   - Introduction (Line 108):   “Integrated broader socioeconomic elements such as employment, urban-rural residence, and wealth index throughout the rationale for the study.”   - Methods:   - Line152: “The study assessed NHI membership at the national level in Indonesia, with a particular focus on understanding the socioeconomic factors influencing participation in the NHI scheme.”   - Line 162: Exposure and Socio-economic Variables   - Line 166: “Residences were classified into two categories: urban and rural. The survey employed the classification standards for urban and rural areas established by the Indonesian Central Bureau of Statistics. Individuals' employment status is classified into two separate categories: employed and unemployed. Participant revenue was ascertained through the utilisation of the wealth index formula in the survey. To determine the wealth index, the survey applied weight to the average of a household's expenditures. During the intervening period, the survey calculated the prosperity index by incorporating various components, including housing, food, healthcare, and essential household expenditures. Furthermore, the survey classified the wealth status into five discrete categories, namely the richest, richer, middle, poor, and poorest [26].”   - Line 177: “The investigation employed six independent variables consisting of education, residence type, age, gender, marital status, employment status, and wealth status. The age of the participants was ascertained using their most recent birthday. The survey delineated gender into two distinct categories, namely male and female. Furthermore, the survey categorised the participants' marital status into three discrete groups: those who had never been married or never in union, those who were still in marital relation or married, and those who were divorced or widowed.” - Results Line 208: “Socioeconomic factors, including education, employment, wealth status, and residence, all showed significant associations with NHI membership. For instance, individuals in urban areas and those in the wealthiest quintile were more likely to be enrolled in the NHI.” |
