# Supplementary material for: National health insurance membership in Indonesia: Do socio-economic elements matter?
Source: PLoS One. 2025 Aug 14;20(8):e0329781. doi: 10.1371/journal.pone.0329781 (PMC12352825; doi:10.1371/journal.pone.0329781)
Supplement: Attachment — Submitted filename: R3_1805_2025_Line 105.docx [file pone.0329781.s006.docx]

article titled *“National Health Insurance Membership in Indonesia: Do Socioeconomic Elements Matter?”*

**General Comments**
This is a well-chosen and timely topic with a comprehensive introduction, thorough analysis, and well-rounded discussion. The study addresses a critical issue in health policy and contributes meaningfully to the understanding of socioeconomic determinants of National Health Insurance (NHI) membership in Indonesia.

**Specific Comments**

**Introduction**

- **Lines 105–106**: *“This study emphasizes the role of education in improving NHI membership among disadvantaged groups, who often face economic and geographic barriers.”*
- **Lines 110–111**: *“This study aims to fill this gap by analyzing how educational attainment influences NHI membership across different socioeconomic strata.”*

**Comment**
Since education appears to be the main independent variable in this study, the analysis should clearly demonstrate its step-by-step contribution to Universal Health Coverage (UHC), especially when introducing other control variables. I recommend that the authors disaggregate the effects of education on NHI membership by levels of disadvantage (e.g., income, geographic location), so it is clear how education impacts each subgroup differently.

**Results**
I find Table 2 somewhat confusing. The purpose of presenting the cross-tabulation of education level across all independent variables is unclear. Instead, it would be more informative if the table were structured to present education level as the first layer of rows, followed by UHC participation as a sub-layer (similar to how sample characteristics are often presented). The leftmost column should list other independent variables for easier comparison and interpretation. This structure would also make Table 2 more directly comparable to Table 1.

Given the study's objective—to assess the contribution of education to UHC participation among poor and disadvantaged groups—it would be more insightful if the results presented a step-by-step analysis of how education contributes to UHC participation within each level of disadvantage.

**Conclusion**
Congratulations to the team on this important research. Keep up the good work!
