# Supplementary material for: National health insurance membership in Indonesia: Do socio-economic elements matter?
Source: PLoS One. 2025 Aug 14;20(8):e0329781. doi: 10.1371/journal.pone.0329781 (PMC12352825; doi:10.1371/journal.pone.0329781)
Supplement: Attachment — Submitted filename: ANSWER TO REVIEWER_PLOSONE_4th_0406.docx [file pone.0329781.s007.docx]

ANSWER TO REVIEWER

| **No** | **Reviewers** | **Reviewer’s Comments** | **Answer to Reviewer** |
| --- | --- | --- | --- |
| 1 | Reviewer 3 | **General Comments** This is a well-chosen and timely topic with a comprehensive introduction, thorough analysis, and well-rounded discussion. The study addresses a critical issue in health policy and contributes meaningfully to the understanding of socioeconomic determinants of National Health Insurance (NHI) membership in Indonesia. | We sincerely appreciate the reviewer’s thoughtful and encouraging feedback. We are grateful that the reviewer finds the topic timely and relevant, and recognises the study’s comprehensive introduction, robust analysis, and balanced discussion. We especially appreciate the acknowledgement of the manuscript’s contribution to understanding the socioeconomic determinants of National Health Insurance (NHI) membership in Indonesia—a subject of growing importance in health policy discourse. |
| 2 |  | **Specific Comments**  **Introduction**   - **Lines 105–106**: *“This study emphasizes the role of education in improving NHI membership among disadvantaged groups, who often face economic and geographic barriers.”* - **Lines 110–111**: *“This study aims to fill this gap by analyzing how educational attainment influences NHI membership across different socioeconomic strata.”*   **Comment** Since education appears to be the main independent variable in this study, the analysis should clearly demonstrate its step-by-step contribution to Universal Health Coverage (UHC), especially when introducing other control variables. I recommend that the authors disaggregate the effects of education on NHI membership by levels of disadvantage (e.g., income, geographic location), so it is clear how education impacts each subgroup differently. | We sincerely thank the reviewer for this constructive comment. Based on the 2^nd^ editor’s observation, and in alignment with the revised title "National Health Insurance Membership in Indonesia: Do Socio-Economic Elements Matter?", we recognise the need to broaden the scope of the study’s objective to include a more comprehensive assessment of multiple socioeconomic determinants, rather than focusing solely on education.  In response:  We have revised the purpose of the study in the Introduction (Lines 105–106 and 110–111) to better reflect the expanded analytical focus on various socio-economic factors such as education, wealth status, employment, residence, and marital status as key variables influencing NHI membership.  We also clarified that the study now explicitly examines the interaction between these variables, particularly how education functions across different levels of disadvantage (e.g., income quintiles and rural/urban settings).  This change enhances the analytical coherence between the study’s objectives, the revised title, and the empirical results.  We believe this adjustment strengthens the contribution of the study by providing a more holistic understanding of the structural factors that influence NHI participation in Indonesia and its implications for achieving UHC.  Revised Paragraphs in the Manuscript:  Previous (Lines 105–106):  This study emphasizes the role of education in improving NHI membership among disadvantaged groups, who often face economic and geographic barriers.  Revised:  This study examines the role of various socio-economic elements—particularly education level, wealth status, employment, and residential location—in shaping NHI membership in Indonesia. These factors are especially relevant among disadvantaged populations that face compounded barriers to health insurance enrolment.  Previous (Lines 110–111):  This study aims to fill this gap by analyzing how educational attainment influences NHI membership across different socioeconomic strata.  Revised:  In line with the revised study title, this research aims to assess how multiple socio-economic factors, including education, interact with structural disadvantages such as poverty and rural residence to influence NHI participation. The study further disaggregates the influence of education within subgroups to better understand its differential effect on advancing universal health coverage (UHC). |
| 3 |  | **Results** I find Table 2 somewhat confusing. The purpose of presenting the cross-tabulation of education level across all independent variables is unclear. Instead, it would be more informative if the table were structured to present education level as the first layer of rows, followed by UHC participation as a sub-layer (similar to how sample characteristics are often presented). The leftmost column should list other independent variables for easier comparison and interpretation. This structure would also make Table 2 more directly comparable to Table 1.  Given the study's objective—to assess the contribution of education to UHC participation among poor and disadvantaged groups—it would be more insightful if the results presented a step-by-step analysis of how education contributes to UHC participation within each level of disadvantage. | We sincerely thank the reviewer for this thoughtful and constructive comment regarding Table 2. We agree that clarity and consistency with Table 1 are essential to support the interpretability of our findings, especially given the study's emphasis on the role of education in mediating UHC participation among disadvantaged groups.  In response:  We have restructured Table 2 to enhance readability and alignment with Table 1. Specifically, the revised table now displays socioeconomic characteristics in the leftmost column, followed by stratification by education level, with NHI membership status (Yes/No) presented as percentages. This format allows for clearer comparison across both education levels and membership status, as suggested.  This format also allows for disaggregated interpretation, enabling a step-by-step understanding of how education interacts with each disadvantage indicator (e.g., income quintile, residence, employment) to shape NHI membership likelihood (Line 2019-230, Page 6-7):  “Table 2 presents the distribution of National Health Insurance (NHI) membership across various socioeconomic and demographic characteristics, stratified by education level. The data reveal a consistent trend in which individuals with higher educational attainment, particularly those with a college-level education, exhibit higher NHI membership rates across all categories, including urban residence, formal employment, marital status, and higher wealth quintiles. Urban residents, for instance, show a notably higher proportion of NHI membership as education level increases, with 85.97% of college-educated individuals insured, compared to 72.01% among those with only primary education. Similar patterns are observed among those in wealthier groups and those employed, suggesting that higher education is correlated with improved access to resources that facilitate NHI enrollment. Statistically significant associations (p < 0.001) and moderate effect sizes (Cramer’s V ranging up to 0.128) underscore the multifactorial influences of education on insurance uptake in Indonesia.”  The revised version (Page 8) facilitates a more intuitive and policy-relevant comparison, particularly regarding how the impact of education on NHI enrolment is influenced by wealth, location, and employment status.  We hope that this improvement better supports the analytical aims of the study and enhances the overall clarity of the presentation.   |
