# Supplementary material for: National health insurance membership in Indonesia: Do socio-economic elements matter?
Source: PLoS One. 2025 Aug 14;20(8):e0329781. doi: 10.1371/journal.pone.0329781 (PMC12352825; doi:10.1371/journal.pone.0329781)
Supplement: Attachment — Submitted filename: rev_5_juli_2025.docx [file pone.0329781.s008.docx]

article titled *“National Health Insurance Membership in Indonesia: Do Socioeconomic Elements Matter?”*

**General Comments**
This is a well-chosen and timely topic with a comprehensive introduction, thorough analysis, and well-rounded discussion. The study addresses a critical issue in health policy and contributes meaningfully to the understanding of socioeconomic determinants of National Health Insurance (NHI) membership in Indonesia.

Specific comments

**Abstract**

Line 33 34 35

**Results (Education Level and NHI Membership):**
The results show that individuals with a junior high school education are **1.214 times more likely** to be National Health Insurance (NHI) members compared to those with only primary education (95% CI: 1.200–1.228). Those with a senior high school education are **1.301 times more likely** (95% CI: 1.286–1.315), and individuals with a college education are **1.916 times more likely** to be NHI members than those with only primary education (95% CI: 1.878–1.954).

**Comment:**
The authors could consider briefly summarizing this pattern, for example: *“Higher education levels are associated with increased likelihood of NHI participation, supporting the notion that education promotes greater access to Universal Health Coverage (UHC).”*

**Introduction (Lines 105–107):**
This study examines the influence of various socio-economic factors—particularly education level, wealth status, employment, and residential location—on NHI membership in Indonesia. These factors are particularly relevant for disadvantaged populations who often face multiple barriers to enrolling in health insurance.

**Comment:**
It appears that the authors aim to compare the relative contributions of education and other independent variables among disadvantaged populations. However, the term *“disadvantaged population”* needs clarification. Does it refer to respondents in wealth quintiles below the fourth (i.e., Q1–Q3)?

**Table 1:**
The table is quite comprehensive. To enhance clarity, it is recommended to add the number (N) and percentage (%) of respondents as the first column. This would help provide a clearer description of the sample characteristics. For instance, although the highest education group shows the highest proportion of NHI participation, their representation in the overall sample is the smallest. In contrast, the lowest education group comprises the largest share of the sample.

**Conclusion**
Congratulations to the team on this important research. Keep up the good work!
